# Food Addiction: Implications for the Diagnosis and Treatment of Overeating

**DOI:** 10.3390/nu11092086

**Published:** 2019-09-04

**Authors:** Rachel C. Adams, Jemma Sedgmond, Leah Maizey, Christopher D. Chambers, Natalia S. Lawrence

**Affiliations:** 1 CUBRIC, School of Psychology, Cardiff University, Maindy Road, Cardiff CF24 4HQ, UK; 2 School of Psychology, College of Life and Environmental Sciences, University of Exeter, Exeter EX4 4QG, UK

**Keywords:** food addiction, overeating, obesity, impulsivity, reward sensitivity, cognitive training, neuromodulation

## Abstract

With the obesity epidemic being largely attributed to overeating, much research has been aimed at understanding the psychological causes of overeating and using this knowledge to develop targeted interventions. Here, we review this literature under a model of food addiction and present evidence according to the fifth edition of the Diagnostic and Statistical Manual (DSM-5) criteria for substance use disorders. We review several innovative treatments related to a food addiction model ranging from cognitive intervention tasks to neuromodulation techniques. We conclude that there is evidence to suggest that, for some individuals, food can induce addictive-type behaviours similar to those seen with other addictive substances. However, with several DSM-5 criteria having limited application to overeating, the term ‘food addiction’ is likely to apply only in a minority of cases. Nevertheless, research investigating the underlying psychological causes of overeating within the context of food addiction has led to some novel and potentially effective interventions. Understanding the similarities and differences between the addictive characteristics of food and illicit substances should prove fruitful in further developing these interventions.

## 1. Introduction

In 2003, obesity was declared a global epidemic by the World Health Organisation [1], and the prevalence of overweight and obesity in both developed and developing countries continues to increase [2,3]. In 2016, 39% of adults were estimated to be overweight and 13% to be obese [4]. Overweight and obesity present a substantial economic burden; in the UK, the total direct and indirect costs are expected to reach £37.2 billion by 2025 [5]. One of the common explanations for the increase in obesity over recent decades is the environment and, in particular, the availability of highly varied, palatable and fattening foods—which have been considered to be addictive [6,7,8,9]. While many individuals manage to resist these temptations and maintain a healthy weight, obese individuals have been shown to have a preference for such energy-dense foods compared to healthy-weight individuals [10,11,12]. The critical question is why some individuals are able to resist overeating while others cannot; what is the evidence for ‘food addiction’ and how can this be used to inform interventions for overeating.

The concept of ‘food addiction’ has been evident in the media and general public for some time and is gaining increasing interest in the scientific literature [13]. There are now numerous reviews discussing the diagnostic, neurobiological and practical aspects of food addiction, with arguments both for and against its utility and validity [14,15,16,17,18,19,20]. This surge of interest comes with the perspective that addiction can be conceptualised as a loss of control over intake for a particular substance or behaviour without the need to focus purely on psychoactive substances [21,22]. The fifth edition of the Diagnostic and Statistical Manual [23] acknowledged this shift in perspective, with the addition of gambling disorder as the first behavioural addiction. Acceptance of this disorder was based on evidence that gambling can produce behavioural symptoms that parallel those of substance addiction and can activate the same neural reward circuits as drugs of abuse [24,25]. There is now a large body of research documenting similar observations for overeating and obesity. Moreover, treatments developed for addictive disorders have also shown some efficacy for the treatment of obesity and overeating. These findings highlight how a model of food addiction may help us to understand elements of overweight/obesity beyond a simple lack of willpower and can also be used to inform effective interventions and policy [26,27,28,29,30].

Food addiction has not yet been recognised in the DSM; however, the similarities between some feeding and eating disorders and substance-use disorders (SUDs) have been acknowledged. These similarities include the experience of cravings, reduced control over intake, increased impulsivity and altered reward-sensitivity. Binge eating disorder (BED) and bulimia nervosa (BN) have been proposed as phenotypes that may reflect these similarities to the greatest extent [31,32,33,34]. Both BED and BN are characterised by recurrent episodes of binge eating in which large quantities of food are consumed in a short time accompanied by feelings of a lack of control, despite physical and emotional distress. Reports of food addiction have been shown to be particularly high amongst these individuals [32,35,36]. Food addiction has also been acknowledged with a standardised ‘diagnostic’ tool—the Yale Food Addiction Scale (YFAS) [37,38]. The YFAS is a questionnaire that parallels the diagnostic criteria for SUDs. The scale has so far been shown to exhibit good internal reliability as well as convergent, discriminant and incremental validity [37,38,39,40].

In this review, we first discuss the DSM-5 diagnostic criteria for SUDs to summarise evidence for food addiction. These criteria are defined as ‘a cluster of cognitive, behavioural and physiological symptoms’ [23]. More specifically, the following categories are considered: impaired control, social impairment, repeated use despite negative consequences and physiological criteria. However, it should be noted that the physiological criteria of tolerance and withdrawal—for which there is less evidence in relation to food—are not necessary for a diagnosis of SUD. The DSM-5 also states that although changes in neural functioning are a key characteristic of SUDs, the diagnosis is based on a pathological pattern of behaviours. Hence, we discuss the diagnostic criteria initially, followed by a review of neurobiological evidence. We then explore the question of how this information can be, and has been, applied to interventions for overeating.

### 1.1. Impaired Control

Taking larger amounts of the substance for longer periods than intended has been cited as one of the most commonly reported symptoms in overweight/obese and BED individuals [41,42]. Excessive and uncontrolled eating also forms the definition of binge eating in BED [23]. Although bingeing can be a planned behaviour, it has been shown that planned binges still result in a greater intake than initially intended [41]. Binge eating has also been documented in non-clinical samples [43,44]; however, in these individuals, occasions of impaired control are more likely to reflect unintentional snacking and excessive portion sizes [8,41,45].

Unsuccessful efforts to restrict food intake are also well documented, with many dieters failing to maintain their diet or even gaining weight in the long term [46,47,48,49,50,51]. In their paper reviewing evidence for refined food addiction (i.e., processed foods with high levels of sugars or sweeteners, refined carbohydrates, fat, salt and caffeine), Ifland et al. [52] report that ‘Every refined food addict reports a series of attempts to cut back on eating. They have used a variety of techniques’ (pg. 521). Curtis and Davis [41] also report similar anecdotes in women with BED who describe avoiding certain trigger foods to control their binges.

The third criterion of time spent obtaining, using and recovering from substance use also translates to BED and BN. These individuals may spend a lot of their time thinking about, engaging in and recovering from binge episodes. As mentioned earlier, bingeing is often a planned behaviour which may require a great deal of effort to purchase and store foods ready for a binge episode [41]. In addition, the criteria for BED emphasise the time spent bingeing, with the number of binge episodes per week determining the severity of the disorder [23]. Moreover, these individuals often experience physical and emotional distress following a binge eating episode. Recovery from food consumption has also been reported in self-identified food addicts with references to feeling sleepy or ‘hung-over’ [52,53].

Although evidence for food addiction directly related to the DSM-5 diagnostic criteria for impaired control is largely anecdotal, there is a considerable amount of empirical evidence for an association between overeating/obesity and impaired control generally. Two aspects of self-regulatory failure that are particularly pertinent in the case of substance use and overeating are impulsivity and reward sensitivity [54,55,56].

#### 1.1.1. Impulsivity

Although impulsivity is a multi-faceted construct, it can be defined broadly as the tendency to think and act without sufficient forethought, which often results in behaviour that is discordant with one’s long-term goals. The role of impulsivity in SUDs is well documented [55,57,58,59,60]. Many studies have reported higher impulsivity levels with increasing substance use across a wide range of questionnaires and behavioural tasks, and for a variety of different substances [61,62,63,64,65,66]. For example, Noël et al. [67] performed a series of behavioural tasks assessing the ability to suppress irrelevant responses (response inhibition) and irrelevant information (proactive interference) in a group of detoxified alcohol-dependent individuals and matched healthy controls. They found a statistically significant group difference for all three tests assessing response inhibition but no differences for proactive interference.

Impulsivity has also been implicated in overeating and obesity [54,68,69,70,71]. Overweight/obese individuals score higher on self-reported [72,73,74] and behavioural measures of impulsivity [75,76,77], whereas those high in self-control have been shown to be less likely to give in to temptation [78,79,80] and are more likely to maintain a healthy diet and engage in physical exercise [81,82,83] Impulsivity scores have also been shown to predict poor food choices [84] and correlate positively with food consumption [85,86,87]. For example, Guerrieri et al. [87] found that, in a sample of healthy-weight women, those with higher impulsivity scores ate more candy during a ‘bogus’ taste test than those with lower impulsivity scores. Churchill and Jessop [88] also showed a predictive relationship between impulsivity and snacking on high-fat foods over a two-week period. Scores on the YFAS have also been associated with various measures of impulsivity, such as motor and attentional impulsivity, mood-related impulsivity and delay discounting [89,90].

#### 1.1.2. Reward Sensitivity

A heightened general sensitivity to reward has also been linked to both substance use and overeating [69,77,91,92,93]. In the food literature, self-report measures of reward sensitivity have revealed associations with BMI, food craving and preferences for foods high in fat and sugar [93,94,95]. Using two behavioural tasks, Guerrieri et al. [69] measured reward sensitivity and response inhibition in children aged 8–10. They subsequently measured food intake in a bogus taste test when the foods were either varied or monotonous. Their results revealed that reward-sensitive children consumed significantly more calories than non-reward sensitive children only when the food was varied. There was no effect of response inhibition on food intake, nor any interaction with variety; however, unlike reward sensitivity, deficient response inhibition was associated with being overweight. The authors suggested that reward sensitivity may play a causal role in overeating, whereas deficient inhibitory control may be more of a maintaining factor. This fits well with findings from a study demonstrating a role of reward sensitivity in the early onset of heroin use and a role of impulsivity in escalating use [92,96].

There is also evidence to suggest that reward sensitivity may decrease with more prolonged or established overeating, with studies showing anhedonia, or hypo-sensitivity to reward, in obese participants [97,98,99,100]. For example, Davis et al. [97] demonstrated that although overweight women were more sensitive to reward than healthy-weight women, those who were obese were significantly *less* reward sensitive than overweight women. Importantly, the earlier mentioned association between reward sensitivity and increased BMI was found in a sample of mainly healthy-weight women, with only 1% classified as obese [93]. Although there is a great deal of evidence to suggest that sensitivity to reward plays a role in substance abuse and overeating, the causal direction of this relationship remains unclear. On the one hand, increasing reward sensitivity may lead to overeating by increasing motivation towards pleasurable activities, such as consuming energy-dense foods that elicit dopamine and opioid activation. On the other hand, decreased reward sensitivity may cause individuals to seek out rewarding activities as a form of ‘self-medication’ in order to boost dopamine functioning (i.e., addictive behaviour is the result of a ‘reward deficiency syndrome’) [101,102]. These two arguments, and the relevant neuroimaging literature, are discussed further below (see the Neurobiological Similarities section below) and in more detail by Burger and Stice [103].

**Figure 1:**
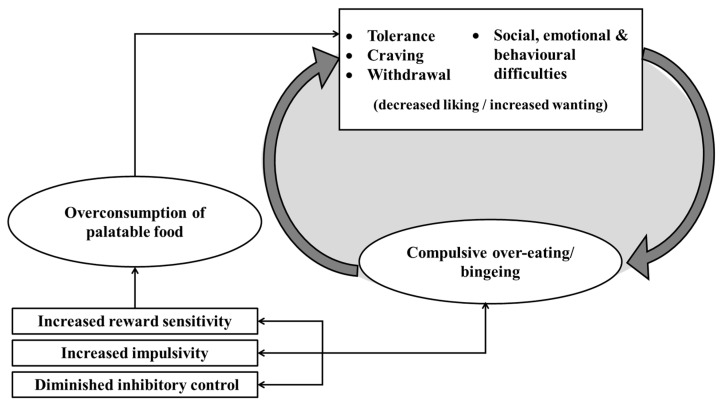
The proposed cycle of ‘food addiction’. Initial vulnerability for the over-consumption of palatable food is marked by increased impulsivity and reward sensitivity, as well as a diminished capacity for inhibitory control. As a consequence of overconsumption, individuals experience tolerance, craving and withdrawal, along with a range of social, emotional and behavioural difficulties such as weight stigmatisation and feelings of guilt and shame. With repeated consumption of these foods, the individual is likely to habituate to the hedonic properties of the food, resulting in reduced enjoyment or liking. These changes are also accompanied by an increased desire or ‘wanting’ for the food [104,105,106,107,108]. In an attempt to relieve these symptoms, the individual ‘self-medicates’ by increasing food consumption, which can result in compulsive or binge eating behaviour, thus creating a cycle of addiction. It should be noted that the extent to which each of these mechanisms is experienced varies considerably across individuals. In particular, initial vulnerability to addiction may be related to individual differences in reward sensitivity, impulsivity and inhibitory control [110,111,112,113].

Burger and Stice [103] offer several theories for how these two causal directions combine to explain obesity. They propose that high sensitivity to reward may initially cause individuals to over-consume palatable foods, but this sensitivity is then modified over time as the brain’s reward system adapts and shows divergent changes in food motivation (‘wanting’) versus hedonic pleasure (‘liking’). According to Robinson and Berridge’s [104,105,106] incentive-sensitisation theory, repeated intake results in an increased incentive value for these foods and their associated cues, which may be subjectively experienced as excessive wanting or craving. Moreover, this theory argues that with repeated presentations of palatable foods, the hedonic pleasure derived from consuming the food will decrease due to neural habituation, while the anticipation of reward increases. Hence, a vicious cycle emerges in which the individual will experience less pleasure from the food (‘liking’), but will simultaneously experience an increased desire (‘wanting’) for the food, driving further food seeking and consumption [107,108,109] (see Figure 1). The experience of intense cravings is the third criterion of impaired control and is another symptom of substance addiction that can be readily applied to overeating and obesity.

### 1.2. Craving

The term ‘food craving’ typically refers to an intense desire to consume a specific food [114,115]. Food cravings appear to be very common with reports of 100% of young women and 70% of young men experiencing a craving for at least one food in the past year [116,117]. The most commonly reported craved food is chocolate, although cravings for carbohydrates and salty snacks are also common [118,119,120,121,122]. The prevalence of food cravings has prompted the development of several standardised questionnaires that measure food cravings with a good degree of internal consistency and construct validity [123,124,125,126,127], including a specific questionnaire just for chocolate (Attitudes to Chocolate Questionnaire) [128]. Recurrent food cravings are of interest in relation to food addiction as they have been associated with binge eating, increased food intake and increased BMI [124,127,129,130,131,132]. Increased reports of food craving have also been demonstrated in individuals who score highly on measures of self-reported food addiction [133,134,135] and those with BED and BN [136,137,138]. Furthermore, just as drug craving is associated with an increased likelihood of relapse [139,140,141], food craving has been linked to poor dieting success [142,143,144].

Further support for the similarity between drug and food craving is evident in the findings of cue-reactivity research. The aphorism that cravings are most likely to occur in the presence of substance-related stimuli has been well documented, with cue-exposure paradigms showing significant effects of drug-related cues on self-reported and physiological measures of craving [145,146,147,148]. Similarly, exposure to food cues has also been shown to increase food cravings [149,150] and a recent systematic review of 45 studies (involving 3292 participants) concluded that ‘food cue-reactivity’ (physiological, neural and subjective reward-related responses to food cues) reliably and prospectively predicts both energy intake and weight gain, particularly over the longer-term, accounting for ~11% (7%–26%) of variance in these outcomes [129]. Food cue-induced craving is especially prevalent among binge eaters and those with BED [151,152] in whom it has been correlated with binge eating frequency and BMI [153]. It is possible, therefore, that certain individuals are more susceptible to cue-induced cravings, and also that this susceptibility may transfer across different substances. Both Mahler and de Wit [147] and Styn et al. [148] found a significant correlation between cue-induced cigarette craving and cue-induced food craving in smokers, suggesting a common mechanism. Cue-induced craving is also believed to strengthen with repeated consumption, fueling the vicious circle shown in Figure 1.

### 1.3. Social Impairment

Overeating and obesity have been associated with poor social functioning, especially among children and adolescents. When assessing quality of life with child and parent-proxy reports, social functioning is significantly lower for obese compared to healthy-weight children and is inversely correlated with BMI [154,155,156]. Poor social functioning in overweight children may be partly due to the overt victimisation and teasing experienced as a direct result of their weight status [157,158]. Hayden-Wade et al. [159] found that the degree of teasing experienced by overweight children was positively correlated with loneliness, an increased preference for isolative activities and a lower preference for social activities. This preference for being alone, along with the emotional difficulty of being victimised, fuels a vicious cycle as these circumstances are likely to promote further overeating and binge-eating—which, in turn, leads to increased weight gain and further teasing [42,160] (see Figure 1).

Weight stigmatisation may also affect interpersonal friendships and romantic relationships in adulthood with reports of discriminatory attitudes and behaviours in occupational [161,162] and romantic settings [158,162,163]. For example, Chen and Brown [164] reported that when making sexual choices about a partner, both male and female college students ranked an obese individual as the least liked. In a study focusing on the psychosocial correlates of food addiction, Chao et al. [165] found that, compared to control participants, those who met the YFAS criteria scored lower on physical, mental and social aspects of health-related quality of life. Social impairments were related to self-esteem, sexual life, public distress and work. Interpersonal problems have also been associated with binge eating—a relationship which is likely to be bidirectional [166,167].

### 1.4. Repeated Use Despite Negative Consequences

It has been noted that due to its increase in prevalence and associated comorbidities, obesity now appears to be a greater threat to the burden of disease than smoking [168]. The physical and psychological effects of overweight and obesity are well documented and include, but are not limited to, depression, an increased risk of diabetes, hypertension, cardiovascular disease and some cancers [169,170,171,172,173,174,175,176,177]. With pervasive warnings regarding the consequences of overeating, from the media, government, and the medical profession, it seems fair to assume that most overweight and obese individuals are aware of the negative outcomes associated with their dietary behaviour [41,52]. Critically, even those who have undergone weight loss treatment often fail to lose weight or gain weight following intervention [46,48,50,51]. Continued overeating also occurs in those who have received bariatric surgery with patients showing continued snacking and poor food choices [178,179]. There is, therefore, considerable evidence to support continued overeating despite negative consequences.

### 1.5. Physiological Criteria

Tolerance to a substance occurs when the same amount of the substance has an increasingly diminished effect with repeated use. This effect usually results in escalated use as the individual increases their dosage in order to recreate the original experience. There is some evidence of food tolerance in animal models of sugar addiction. Rats given intermittent and excessive access to sugar solution increase their intake significantly over time, and this is accompanied by neurochemical changes that are similar to those seen in drug abuse [180,181]. In humans, there is some indication that tolerance to sugar may occur in the first few years of life. The effectiveness of sucrose as an analgesic in young infants is reported to diminish after 18 months of age as sugar consumption increases [182,183,184,185]. The possibility of such early tolerance to palatable foods and the methodological difficulties of diet restriction in humans makes finding empirical evidence of tolerance in adults difficult and unlikely. However, statistics indicating increased consumption and portion sizes for these foods provide indirect evidence of tolerance to high-fat/high-sugar foods at a population level [52,186], and also at an individual level based on anecdotal reports. For example, Pretlow [42] found that 77% of overweight poll respondents reported eating more now than when they originally became overweight. Furthermore, in response to a follow-up question asking why they believed that they ate more, 15% indicated that they were less satisfied by food. Hetherington et al. [109] also found that when participants were provided with chocolate for three weeks, they increased their intake over time while simultaneously reporting a reduction in food liking.

Withdrawal is the second physiological criterion for substance abuse and is defined by the presence of physical or psychological symptoms in response to substance deprivation, or the use of the substance in order to relieve these symptoms. Evidence of withdrawal has also been found in the aforementioned animal models of sugar addiction. Under conditions of sugar deprivation, these animals show withdrawal symptoms similar to those seen with morphine and nicotine withdrawal, including physical symptoms of teeth chattering, forepaw tremor, head shaking and reduced body temperature [187,188] as well as increased aggression [189] and anxiety [190]. There are also anecdotal reports of withdrawal-like symptoms in humans, including persistent cravings and negative affects when attempting to reduce food intake [42,191], as well as the tendency to eat to avoid the emotional symptoms associated with withdrawal such as fatigue, anxiety and depression [52]. Using the YFAS, withdrawal symptoms (such as agitation, anxiety, or other physical symptoms) have been reported in up to 50% of individuals with obesity and BED [35].

## 2. Neurobiological Similarities between Palatable Foods and Drugs of Abuse

Just as altered brain functioning has been reported in SUDs, overeating and obesity have also been associated with changes in the neural processing of the motivational properties of food. This includes changes in systems coding the hedonic and rewarding aspects of the substance, as well as the systems involved in controlling these motivations [103,192,193,194]. Volkow and colleagues [195,196,197,198,199] have proposed a common model for addiction and obesity that involves two neural circuits that are both modulated by dopamine—increased reward sensitivity and diminished inhibitory control [70].

### 2.1. Neurobiology of Reward Sensitivity

Addictive drugs directly affect the mesolimbic dopamine system (MDS), which is thought to mediate the processing of motivational salience, pleasure and reward [200]. Animal studies have shown that, similar to drugs of abuse, palatable foods are capable of triggering dopamine release in the nucleus accumbens (NAc) and ventral tegmental area (VTA) [181,201,202,203]. Furthermore, activity in the MDS has been linked to the amount of food ingested and its rewarding properties [204,205]. However, distinct patterns of neuronal firing in the NAc to food and illicit substances have also been reported [206,207]. Increased activation of this reward system has also been shown in human participants during the presentation of food cues and meal consumption [96,208,209,210,211]. For example, Stoeckel et al. [212] demonstrated that when viewing images of high-calorie foods, obese women showed significantly greater activation in a number of regions associated with reward, compared to healthy-weight women. Obese participants have also demonstrated increased responsivity to food in gustatory and somatosensory regions [213,214], suggesting a heightened sensitivity to palatable food that may contribute to overeating and obesity.

Although an increased sensitivity to reward may initially drive individuals to consume calorific foods, it has been speculated that compulsive eating may develop as the pleasure derived from these foods diminishes with increased tolerance (see Figure 1). It has been argued that, just as with drugs of abuse, the chronic consumption of such rewarding foods may cause the downregulation of dopamine receptors in order to compensate for their overstimulation [215,216,217]. Decreased striatal dopamine receptor availability has frequently been observed in individuals with substance addictions [218,219,220,221,222], whereas increased receptor availability has been shown to have a protective role against alcoholism [223,224]. It has also been shown that striatal D2 receptor availability is significantly lower in severely obese individuals compared to controls and is significantly and negatively correlated with BMI [99,100].

**Figure 2:**
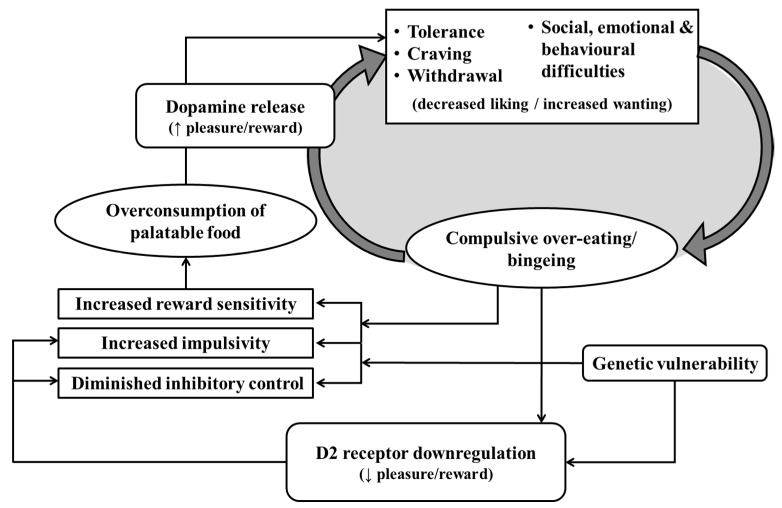
The proposed cycle of ‘food addiction’ including the role of dopamine. When palatable food is consumed, the brain releases the hormone dopamine (alongside other neurotransmitters such as opioids). Over time, this increase in dopamine leads to the downregulation of dopamine receptors, causing individuals to experience a reduction in pleasure during palatable food consumption. This decrease in pleasure, combined with symptoms of tolerance, craving, withdrawal and other social, emotional and behavioural difficulties, results in the individual engaging in compensatory behaviour by increasing food consumption. As a consequence, food consumption may become compulsive, thus creating a cycle of food addiction.

It has been argued, therefore, that a reduction in dopamine receptor availability may subsequently cause or exacerbate overeating as a form of ‘self-medication’ in which the individual attempts to compensate for a diminished experience of reward [100,225,226,227] (see Figure 2). For example, Geiger et al. [228] found that rats fed on a cafeteria-style diet showed reduced baseline levels of mesolimbic dopamine activity. This activity was stimulated by cafeteria foods but not by their regular chow, thus suggesting that a preference for palatable food may develop as a consequence of its ability to increase dopamine release compared to other, less palatable, foods. Animal studies have also demonstrated causal effects of D2 receptor agonists and antagonists on overeating. The administration of D2 antagonists has been shown to increase meal size, meal duration and body weight, whereas treatment with D2 agonists can reduce hyperphagia and prevent weight gain [229,230,231]. The effects of such pharmaceutical interventions in humans, however, have been fairly mixed. The use of antipsychotic medication which blocks D2 receptors is typically associated with weight gain [232] and some D2 agonists have been found to reduce body weight [233]. A recent trial, however, found no effect of the dopamine agonist cabergoline on preventing weight regain [234,235] and there is some evidence that D2 agonists can promote weight gain in patients with anorexia nervosa [236]. More encouragingly, studies with gastric bypass patients have demonstrated increased D2 receptor availability following weight loss, indicating that the effects of overeating on dopamine receptor downregulation may be reversible [237,238,239].

### 2.2. Neurobiology of Inhibitory Control

Dopamine receptor availability in obese individuals has also been shown to correlate positively with metabolism in prefrontal regions involved in inhibitory control (specifically the dorsolateral prefrontal cortex [DLPFC], medial orbitofrontal cortex [mOFC] and anterior cingulate gyrus, as well as the somatosensory cortices) [99]. Similar findings have been observed in healthy-weight participants, who demonstrated a positive correlation between dopamine receptor availability and inhibitory control performance on the stop-signal task [240]. Volkow et al. [99] hypothesised that altered dopamine functioning may play a role in overeating not only through altering the rewarding properties of food but also by reducing inhibitory control. A significant negative correlation between BMI and prefrontal activity has also been reported [75,241,242] along with reduced prefrontal activation following a meal in obese men and women [243,244,245]. Conversely, successful dieting has been positively associated with frontal activation [246,247,248,249].

In a study of healthy women, Lawrence et al. [96] reported an association between food cue reactivity in the NAc and later snack consumption [117]. They also found that this reactivity was associated with increased BMI for individuals who reported low self-control. The authors proposed a ‘dual hit’ of increased reward motivation and poor self-control in predicting increased food intake [250]. Similarly, reductions in frontal grey matter volume have also been linked to increased BMI, poor food choices and related deficits in executive functioning [251,252,253,254,255,256,257,258]. These findings are reflective of a growing literature on the cognitive dysfunction associated with drug abuse and obesity, although research indicates that the causal relationship is bidirectional [76,259,260,261,262,263].

Although it has been hypothesised that overeating is initially caused by a hyper-responsive reward circuitry and maintained by the subsequent degradation of this system [103], there is also evidence to suggest that some individuals may be genetically vulnerable to an impaired capacity for reward and inhibitory control. Genetics studies have revealed that both drug users and obese individuals have a significantly greater prevalence of the *Taq*I A1 allele polymorphism which can cause a 30%–40% reduction in striatal D2 receptors [213,264,265,266,267,268,269]. In addition, this polymorphism has been associated with behavioural measures of impulsivity and low reward sensitivity [270,271,272]. It has also been linked to low grey matter volume in the anterior cingulate cortex (ACC) [273], an area which is believed to be involved in executive control and reward expectancy [240,274,275], and has been shown to be active during resistance of cigarette craving [276]. Together these findings demonstrate that overeating and SUDs may share a common neurobiological mechanism involving altered dopamine functioning that subsequently disrupts mechanisms involved in reward sensitivity and inhibitory control.

Our review, considering each of the DSM-5 criteria for SUDs in isolation, suggests that there is considerable evidence for food addiction. Whether an individual meets clinical diagnostic criteria under an SUD model, and the severity of the disorder, however, is dependent on an individual presenting a number of symptoms (mild: two to three symptoms; moderate: four to five symptoms; severe: six or more symptoms). Studies utilising the YFAS (which uses diagnostic criteria for SUDs) have certainly suggested that a substantial proportion of the general population meet the diagnostic cut-off for food addiction (15%–20%), with approximately 11% of the population being classified as ‘severe’ [38,276]. The prevalence of food addiction in those with BED and BN has been reported as much higher, with estimates of 92% for BED and 96%–100% for BN [32,277,278]. Acknowledging the potential prevalence of food addiction, we next discuss a range of treatments for overeating that have been informed by the similarities between SUDs and overeating.

## 3. Treatment Implications

One of the greatest potential advantages of identifying the similarities between substance addictions and overeating is the development of effective interventions. The standard approach to weight loss, involving maintaining a healthy diet and physical exercise, is often associated with poor adherence rates and overall weight gain [46,47,48,49,50,51,279]. One possible reason for the ineffectiveness of dieting is that it is treating the outcome of overeating and not the underlying cause. Approaches that target increased impulsivity and reduced self-control may have more success. For example, Hall, Fong, Epp and Elias [280] showed that executive function on the go/no-go task (a measure of response inhibition) predicted unique variance for dietary behaviour and physical exercise, and also moderated the association between intentions and behaviour [117,281]. This suggests that individuals who are more capable of controlling their impulsive actions are more likely to successfully meet their goals. This also implies that techniques to improve such abilities may prove to be effective tools for aiding weight loss.

### 3.1. Cognitive Interventions

Increased motivation for illicit substances has been associated with several cognitive biases including attentional biases [282,283,284,285,286,287], approach biases [283,284,288,289,290] and affective biases [291,292,293,294]. One method for reducing this motivation, therefore, has been to use training tasks that are designed to reduce these cognitive biases, and recently, these training tasks have been explored as potential interventions for overeating.

Heightened attentional biases towards food have been demonstrated across various populations, including those with disordered eating patterns [295,296,297,298,299,300] and those who are overweight or obese [301,302,303,304]. Just as the addiction literature has explored whether attentional biases can be manipulated to reduce substance intake, this approach has also been explored with food consumption, although with mixed results [305,306,307,308,309]. Hardman et al. [305] trained undergraduate students on the visual probe task to either attend or avoid images of cake and stationery. They found a modest increase in attentional bias for the attend-cake group but no effects of bias training, for either group, on hunger or food consumption, suggesting that any attentional biases with food may be particularly difficult to modify. Using a female-only sample, Kemps et al. [306] demonstrated significant effects of a similar dot-probe training on attentional bias and food consumption. Effects on attention were found to generalise to novel pictures, however, effects on food intake were specific to the trained food and were undermined by participants consuming more of an equally unhealthy novel food. More recent results hold some promise for attentional bias modification, indicating that training can be used to decrease immediate calorie consumption in overweight and obese women [310] and can increase the consumption of healthy foods [311]. Multiple training sessions have also demonstrated that effects can persist beyond the period of training for up to a week [312].

There is also a small body of evidence demonstrating an approach tendency towards food for individuals with disordered eating [295,313,314], high trait food craving [315] and those who are overweight or obese [316,317,318]. For example, Veenstra and de Jong [313] showed that those who scored highly on a measure of dietary restraint (a measure for the chronic, cognitive limitation of food intake) were significantly faster to move a manikin towards than away from images of food. Using a different measure of approach bias, Kemps, Tiggemann, Martin and Elliott [319] also found that participants who liked chocolate were significantly faster at pairing images of chocolate with approach words compared to avoid words. Furthermore, they also demonstrated that participants who were trained to pair images of chocolate with either approach or avoid words increased and decreased their approach bias, respectively. The approach group also demonstrated a significant increase in chocolate cravings. Although the avoid group showed a decrease in reported craving, this finding was not statistically significant from baseline [320]. Similar training protocols have also been shown to be effective at reducing action tendencies towards high-calorie foods and reducing trait- and cue-induced craving in participants with subclinical eating disorders [321].

The use of addictive substances can also be motivated by the positive affect associated with them; therefore, reducing such an affective bias should discourage their use. Studies using evaluative (or affective) conditioning have shown initial promise. Here, the evaluation of a conditioned stimulus (CS) can be modified by consistently pairing it with a valenced unconditioned stimulus (US) [322,323]. In the food literature, negatively valenced stimuli are typically used to reduce the implicit liking of unhealthy foods [324,325,326,327,328]. Further, evaluative conditioning has been found to lead to more favourable food choices in some studies. For example, Walsh and Kiviniemi [329] found that participants were three times more likely to select fruit over a granola bar after receiving evaluative conditioning training where positive (relative to negative or neutral) words and images were paired with images of fruit. Similarly, Hollands et al. [325] showed that participants were more likely to select fruit over an unhealthy snack when snack images were repeatedly paired with negative body images compared to a blank screen. Interestingly, this effect was moderated by implicit attitudes towards the snack foods; participants with more favourable attitudes at baseline showed the greatest change in subsequent behaviour. Although these studies have involved healthy participants, this latter finding, in particular, suggests that evaluative conditioning may be an appropriate intervention for those with disordered eating who show strong preferences for unhealthy foods. However, the effects of evaluative conditioning training on reducing unhealthy food consumption are currently unclear. To date, only a limited number of studies have included post-intervention follow-up, and while some have found reduced consumption in the week following training [330], others have failed to show immediate effects [327,328,330]. It is likely that training effects are dependent on baseline strength of liking for specific foods [325,330], the specificity of the US used [331], and awareness of the CS-US contingencies [332,333,334]. To establish the therapeutic benefits of evaluative conditioning training, studies in overweight and disordered eating groups are required.

Another approach to cognitive training is to reduce such biases indirectly through tasks such as response inhibition training. Response inhibition refers to our ability to interrupt or override impulsive reactions in accordance with new information, and plays a key role in goal-directed behaviour [335,336,337,338,339,340]. Deficient response inhibition has been linked not only to the use of different addictive substances [70] but also to the severity of use [61,64,341], poor treatment outcomes [342] and likelihood of relapse [343]. Houben and Wiers [344] have also shown that positive implicit attitudes towards alcohol are only related to alcohol consumption when inhibitory control is low. These results suggest that an increased ability to inhibit responses may enable an individual to exert self-control over their behaviour, even when they possess strong implicit preferences [117].

Similar findings have also been replicated with overeating and obesity. Obese individuals have been shown to demonstrate less efficient response inhibition than their healthy-weight counterparts [69,251,345,346] and poor inhibitory control has been associated with increased unhealthy food consumption [86,347,348], high BMI [75,85,349,350], food cravings [351], unhealthy food choices [84,352] and binge-eating [353]. Moreover, as in the addiction literature, inhibitory control has also been shown to interact with implicit attitudes towards food, thus indicating that effective response inhibition may play a protective role against strong implicit preferences for unhealthy foods [80,117,250].

Simple tasks designed to train response inhibition to relevant cues or contexts have been shown to reduce gambling behaviour and alcohol consumption [354,355,356,357,358,359]—although available evidence suggests that the longevity of such effects may be limited [360]. These training tasks have also been adapted to train response inhibition to food stimuli and are showing encouraging effects across a range of eating-related behaviours including food consumption [361,362,363,364,365], food choices [365,366,367,368,369,370,371] and even weight loss [372,373,374,375]. For example, Lawrence et al. [376] trained participants to inhibit their responses towards either images of unhealthy snack foods (active group) or non-food items (control group). After four training sessions, they found that, compared to the control group, individuals in the active group showed reduced energy intake (220 kcal less per 24 h food diary) and reduced liking for unhealthy foods. Furthermore, participants in the active group showed significant weight loss; showing objectively measured weight loss of 0.7 kg after 2 weeks and self-reported weight loss of more than two kilograms after a six month follow up (2.66% decrease).

The cognitive training paradigms discussed above show promise but are currently in the early phases of testing. Before such training methods can be taken forward to clinical trials, researchers should further explore the effects of different experimental protocols with the aim of developing the most effective training techniques. One aspect of training that is likely to be important in determining successful behavior change is training performance. For example, the proportion of successful inhibitions on an inhibition training task and accuracy during attentional bias training have both been shown to moderate efficacy [308,375]. To establish whether effects can be long-lasting, we need to consider repeated testing sessions, personalised training stimuli and combining training techniques to simultaneously reduce cognitive biases and increase executive control [312,369,377,378]. Understanding the mechanisms that underlie such effects could also prove crucial. For example, the effects of inhibition training on food consumption may be due to the devaluation of inhibited stimuli. Several studies have shown that repeatedly pairing a stimulus with the inhibition of a response can reduce how much the image is liked or how attractive it was perceived to be [371,376]. Such devaluation could be the result of action conflict or inherent links between avoidance and aversion [371,379,380,381,382]. Designing interventions that promote automatic associations between stimuli and action tendencies may, therefore, prove fruitful, especially if training is performed accurately, personalised and delivered across multiple sessions. Combining cognitive training tasks with prefrontal brain stimulation is another avenue worthy of investigation. Brain stimulation methods have the potential to augment learning effects [383] and can also be used to reduce food consumption and craving in isolation; these methods are discussed below.

### 3.2. Neuromodulation Interventions

Non-surgical brain stimulation techniques have also been explored for their potential benefits in reducing craving and addictive behaviours by altering neural activity and increasing dopamine [384,385,386,387,388]. The most commonly applied stimulation methods are transcranial magnetic stimulation (TMS) and transcranial direct current stimulation (tDCS). These methods are used in awake participants and are generally considered to be safe when administered within recommended guidelines [389,390,391,392,393,394].

The use of TMS involves the delivery of electromagnetic pulses that penetrate the skull to induce electric current in the underlying cortex and cause short-term changes in cortical excitability. The modulation of cortical excitability can last beyond the period of stimulation by delivering trains of pulses, a technique known as repetitive TMS (rTMS) [395]. When applied to the DLPFC, rTMS has been shown to effectively reduce cravings for cigarettes, alcohol and drugs of abuse, especially when applied for multiple sessions [396,397,398,399,400]. The DLPFC is an area involved extensively in inhibitory control [401,402,403,404] and stimulation of this region may act to boost self-control, potentially by increasing dopamine release in the caudate nucleus [405,406].

Reductions in substance craving have also been demonstrated with stimulation of the DLPFC using tDCS [114,407,408,409,410,411,412]. The use of tDCS involves the application of a weak (typically 1–2mA) direct electrical current to the scalp via a pair of electrodes. The effect of tDCS on brain activity is dependent on the stimulation polarity; anodal stimulation is thought to increase cortical excitability by neuronal depolarisation whereas cathodal stimulation is believed to decrease excitability by hyperpolarising neurons [413,414,415,416,417,418]. Long-lasting effects on resting membrane potential have been shown with longer stimulation durations, for example, 13 min of anodal tDCS has been shown to increase motor cortical excitability for up to 90 min [419]. Compared to TMS, tDCS is a weaker form of stimulation with fewer incidental artefacts and is considered to be safer and more appropriate for reliable double-blinding [387,420,421]. It is also thought that tDCS can be used to potentiate learning [422], and may effectively enhance the effects of the aforementioned cognitive interventions [378,423].

These stimulation methods are currently being investigated for their potential to reduce food craving and consumption [424,425,426,427,428,429]. Using rTMS to the left DLPFC, Uher et al. [430] found an increase in cue-induced craving for palatable foods in the group who experienced sham stimulation but not the active group. However, no effect was found on ad-libitum food consumption, although this result may have been due to the limited time period (5 min) causing participants in both groups to consume a large amount of calories. Using a similar methodology, Van den Eynde et al. [431] demonstrated an increase in craving scores in the sham group, but a decrease in craving scores for the active group in a sample of participants with bulimic-type eating disorders. In addition, active rTMS was associated with a reduction in binge-eating episodes in the following 24 h period. However, blinding was only partially successful in this study with most participants correctly guessing whether they were receiving active or sham rTMS. In a later study, Barth et al. [432] used a within-subjects design with an improved sham condition in which they matched the perceived pain of active rTMS with scalp electrodes. They found an equal reduction in cravings for both conditions and attributed this effect to the experience of pain rather than prefrontal stimulation.

As mentioned, tDCS is believed to involve a more appropriately matched sham condition, especially when participants receive active stimulation for a short initial period [420,421]. When stimulating the DLPFC bilaterally using tDCS, Fregni et al. [433] found a significant increase in cue-induced craving, measured before and after stimulation, in the sham condition and a significant reduction when participants received anodal right/cathodal left stimulation. Compared to the sham condition, active stimulation was also associated with a reduction in food intake during an ad-libitum eating phase. Although the authors did not assess blinding in this study, they did report equal occurrences of mild adverse effects across conditions. Using the same montage, Goldman et al. [424] and Lapenta et al. [426] also found the same reduction in food craving, and an early meta-analysis revealed a medium effect-size favouring active over sham stimulation in the reduction of cravings [434]. However, as the number of studies utilizing tDCS in the exploration of its effects on food craving has increased, evidence of efficacy has weakened. A more recent meta-analysis, including eight experiments, found no effect of tDCS on food craving [386], and subsequent research, including a large pre-registered experiment, has also failed to replicate findings for both food craving and consumption [435,436].

Another neuromodulation intervention, which is worthy of a brief mention and gaining in popularity for the treatment of SUDs, is real-time fMRI (rt-fMRI) neurofeedback training. Neurofeedback training involves providing participants with feedback of their neural response to certain cues and instructing them to increase or decrease their response, so that they may gain volitional control over specific brain regions. In the treatment of SUDs, neurofeedback training typically involves increasing activity in control regions, such as the prefrontal cortex, or decreasing activity in regions associated with craving, such as the ACC. For example, it has been shown that decreasing activity in the ACC with rt-fMRI neurofeedback is significantly correlated with decreased nicotine craving in smokers [437,438,439]. Using a similar technique with electroencephalography (EEG) has also shown improvements in cravings, drug use and treatment outcomes for a range of different substances [440,441,442,443]. Although in its early days, the application of neurofeedback training to food consumption and obesity has already been proposed [444,445,446]; recent findings have also suggested that neurofeedback may be another method of decreasing activity in motivation- and reward-related regions [447] and increasing activity within critical prefrontal regions such as the DLPFC [448,449].

### 3.3. Therapeutic Interventions

Therapeutic interventions such as Overeaters Anonymous and cognitive behavioural therapy have taken a more holistic approach to the treatment of obesity. Overeaters Anonymous (OA) is based directly on the 12-step programme developed by Alcoholics Anonymous. The OA organisation promotes the central belief that obesity is a symptom of ‘compulsive overeating’, which is an addictive-like illness with physical, emotional and spiritual components [450]. Individuals are required to acknowledge that compulsive overeating is beyond their willpower to overcome and, therefore, they must attempt to control their intake by avoiding certain foods and surrendering to a ‘higher power’. Just like Alcoholics Anonymous, OA involves group meetings for individuals to share their feelings and experiences. Although the way in which this programme influences outcomes is unclear [53], the group meetings may act to alleviate feelings of isolation and instead foster a sense of community. As discussed earlier, due to the feelings of shame and guilt and the weight teasing experienced, overweight and obesity are associated with a preference for isolative activities [159]. This social isolation can subsequently exacerbate overeating, creating a vicious cycle [42,160]. It is possible, therefore, that OA acts to break this cycle by providing a supportive and encouraging social environment. However, due to the anonymous nature of OA, there has been little research conducted on its efficacy and it is not understood exactly how OA affects overeating and the extent to which it may do so.

Cognitive behavioural therapy (CBT), on the other hand, is a therapeutic approach which is extensively informed by research. CBT requires patients to critically evaluate the thoughts, feelings and behaviours that result in maladaptive behaviour and then modify them through therapy. This therapy allows patients to recognise potential triggers and develop appropriate coping strategies. CBT interventions have been effective in the treatment of substance addictions [451] and have also demonstrated their potential in the treatment of obesity [452,453] and BED [453,454,455,456]. However, it has been argued that the success of treating overeating and BED with CBT refutes the food addiction model [30]. The food addiction model applied in OA requires complete avoidance of so-called trigger foods, thereby acting to increase dietary restraint, whereas a reduction in dietary restraint has been shown to moderate the increased effectiveness of CBT on binge eating in a sample of patients with BN [457]. The focus of CBT is to replace dysfunctional eating with more normalised eating behaviour, therefore, favouring moderation and flexibility rather than absolute restraint.

## 4. Conclusions

As the prevalence of obesity continues to increase and traditional weight loss methods appear to be largely unsuccessful, researchers and clinicians have begun to consider the addictive potential of food. There is a substantial body of evidence demonstrating the similarities between addictive drugs and food on reward and control pathways in the brain and subsequent behaviour such as craving and impulsivity. There is also limited evidence to indicate that in some circumstances, overeating meets the physiological criteria of substance dependence, although more research is necessary to determine the validity of these symptoms in human participants. More research is also required for other behavioural criteria such as social impairment and repeated use despite negative consequences, as the evidence to date is largely anecdotal. However, meeting the physiological criteria for addiction is not necessary for a DSM diagnosis, and as food is a legal substance, just like caffeine, tobacco and alcohol, not all criteria associated with SUDs [23] readily translate to food addiction. Nevertheless, the criterion of withdrawal in SUDs has been associated with clinical severity and the number of symptoms that an individual endorses is used to determine the disorder’s overall severity [23].

With a number of these criteria having a limited application to food addiction, a clinical diagnosis appears unlikely in most cases of overeating; however, using the YFAS, it has been estimated that approximately 11% of the general population meet the criteria for a ‘severe’ food addiction [38]. It should also be made clear that the concept of food addiction does not equate with obesity. Obesity is a multifactorial condition determined by genetic, environmental, biological and behavioural components. For the majority of cases, obesity is caused by a steady increase in excess energy intake and it is not characterised by a compulsive drive for food consumption. Instead, it is thought that the concept of food addiction applies most appropriately to individuals with BED and BN [31,32,277,278].

Despite there being considerable parallels between substance use and compulsive overeating, there is still some concern regarding the use and validity of the term ‘food addiction’, which is unlikely to apply to the majority of cases [17]. There is also concern over the use of such terminology in the wider social context and whether the term may do more harm than good. While most people would believe that an addiction model reduces individual responsibility, it has also been argued that attributing the problem to a minority of individuals also reduces corporate responsibility [28,458]. As the majority of the population would not be considered ‘food addicts’, there would be less pressure for the food industry to reduce marketing or to promote healthier alternatives. Likewise, any environmental interventions to reduce access and availability may also seem less critical with a food addiction model.

There are also implications of such terminology for the diagnosed individual. Obesity is already associated with significant social stigmatisation [157,158,159,161,162,163,164] and an additional ‘addict’ label, which may invoke stereotypes of a person who is untrustworthy and inferior [459], may only serve to heighten the problem [460,461,462]. DePierre et al. [460] found that when an individual was labelled as an ‘obese food addict’ they were more stigmatised than when they received either label in isolation (‘food addict’ or ‘obese’). However, a study investigating the effect of an addiction model on public perceptions found that it actually reduced stigma, blame and perceived psychopathology [463,464], suggesting that it may be beneficial in reducing weight-related prejudice. The ‘addicted’ individual described in the study was viewed as being less at fault for their weight. Although, it is unclear whether the fault then lies with the individual’s biology (i.e., certain individuals are prone to becoming ‘food addicts’) or the industry that continues to promote potentially addictive foods. Although it is almost certain to be a combination of both entities, demonstrating that certain foods can be addictive should increase corporate responsibility and pressure on the food industry to regulate the availability, advertising and nutritional content of such palatable foods [9,458].

Despite these issues and concerns, it has also been acknowledged that for some individuals, ‘food addiction’ may be the most appropriate diagnosis for their symptoms and it may help to inform their treatment [34]. The available evidence suggests, therefore, that some individuals *are* capable of experiencing an addictive-type relationship with food, although the majority of individuals who compulsively overeat are unlikely to receive such a diagnosis. Considering the underlying causes of impulsive overeating has also led to the development of some exciting and potentially effective interventions. While there are differences between the addictive characteristics of food and illicit substances, there are many parallels that should not be ignored. These parallels have contributed greatly to our current knowledge of compulsive overeating and potential treatments. Both the similarities and differences should encourage more research, which is necessary to determine the extent and potential impact of such a disorder. Until then, the idea of ‘food addiction’ is expected to remain hotly debated [14,19,20].
